# Solving the spike feature information vanishing problem in spiking deep Q network with potential based normalization

**DOI:** 10.3389/fnins.2022.953368

**Published:** 2022-08-25

**Authors:** Yinqian Sun, Yi Zeng, Yang Li

**Affiliations:** ^1^Research Center for Brain-Inspired Intelligence, Institute of Automation, Chinese Academy of Sciences, Beijing, China; ^2^School of Future Technology, University of Chinese Academy of Sciences, Beijing, China; ^3^School of Artificial Intelligence, University of Chinese Academy of Sciences, Beijing, China; ^4^National Laboratory of Pattern Recognition, Institute of Automation, Chinese Academy of Sciences, Beijing, China; ^5^Center for Excellence in Brain Science and Intelligence Technology, Chinese Academy of Sciences, Shanghai, China

**Keywords:** brain-inspired decision model, SDQN, reinforcement learning, potential normalization, spiking activity

## Abstract

Brain-inspired spiking neural networks (SNNs) are successfully applied to many pattern recognition domains. The SNNs-based deep structure has achieved considerable results in perceptual tasks, such as image classification and target detection. However, applying deep SNNs in reinforcement learning (RL) tasks is still a problem to be explored. Although there have been previous studies on the combination of SNNs and RL, most focus on robotic control problems with shallow networks or using the ANN-SNN conversion method to implement spiking deep Q networks (SDQN). In this study, we mathematically analyzed the problem of the disappearance of spiking signal features in SDQN and proposed a potential-based layer normalization (pbLN) method to train spiking deep Q networks directly. Experiment shows that compared with state-of-art ANN-SNN conversion method and other SDQN works, the proposed pbLN spiking deep Q networks (PL-SDQN) achieved better performance on Atari game tasks.

## 1. Introduction

Inspired by biological brain neurons, the spiking neural network uses differential dynamics equations and spike information encoding methods to build computing node models in neural networks (Maass, 1997). The traditional artificial neuron models, such as Perceptron and Sigmoids, sum the inputs and pass them through a non-linear activation function as model output. Unlike ANNs, the spiking neurons accept signals from presynaptic inputs with a particular synapses model. They then integrate the post-synaptic potential, firing a spike when the somatic potential exceeds a threshold. The neuron potential is reset when the spike is released to prepare for the next integrate-and-fire process. According to the complex structure and dynamic characteristics of biological neurons, the spiking neuron model has many forms, including the leaky integrated-and-fire (LIF) model, Izhikevich model, Hodgkin-Huxley, and spike response model.

Spike neural networks can be applied to different domains of pattern information processing. SNNs have achieved competitive performance on many tasks compared with ANN. Spiking Resnet is trained for image classification (Fang et al., 2021), and Spiking-YOLO (Kim et al., 2020) uses the ANN-SNN conversion method to implement faster and more efficient object detection. Brain visual pathway-inspired spiking neural networks process image features with biologically plausible computing method (Hopkins et al., 2018). CRSNN (Fang and Zeng, 2021) implemented causal reasoning with SNN and Spike-Timing-Dependent Plasticity (STDP). QS-SNN (Sun et al., 2021) takes advantage of a spatio-temporal property of spike trains and processes complement information with spiking rate and phase encoding. In addition, Zhao et al. (2018) and Cox and Witten (2019) implement basal ganglia-based SNNs models in many decision-making tasks. Besides, using neural networks to decode neuronal spike trains and activity enables an understanding of how the brain processes sensory and behavioral signals. Deep neural networks decoder are used to reconstruct pixel-level image features from two-photon calcium neural signals (Zhang et al., 2022). Additionally, Xu et al. (2022) proposed a DSPD framework to reconstruct multi-modal sensory information from neural spike representations. The neuromorphic hardware based on SNNs, such as TrueNorth (Merolla et al., 2014), SpiNNakers (Furber et al., 2014), and Loihi (Davies et al., 2018) reduces the energy consumption by thousands of times than chips based on traditional computing architecture.

**Figure 1:**
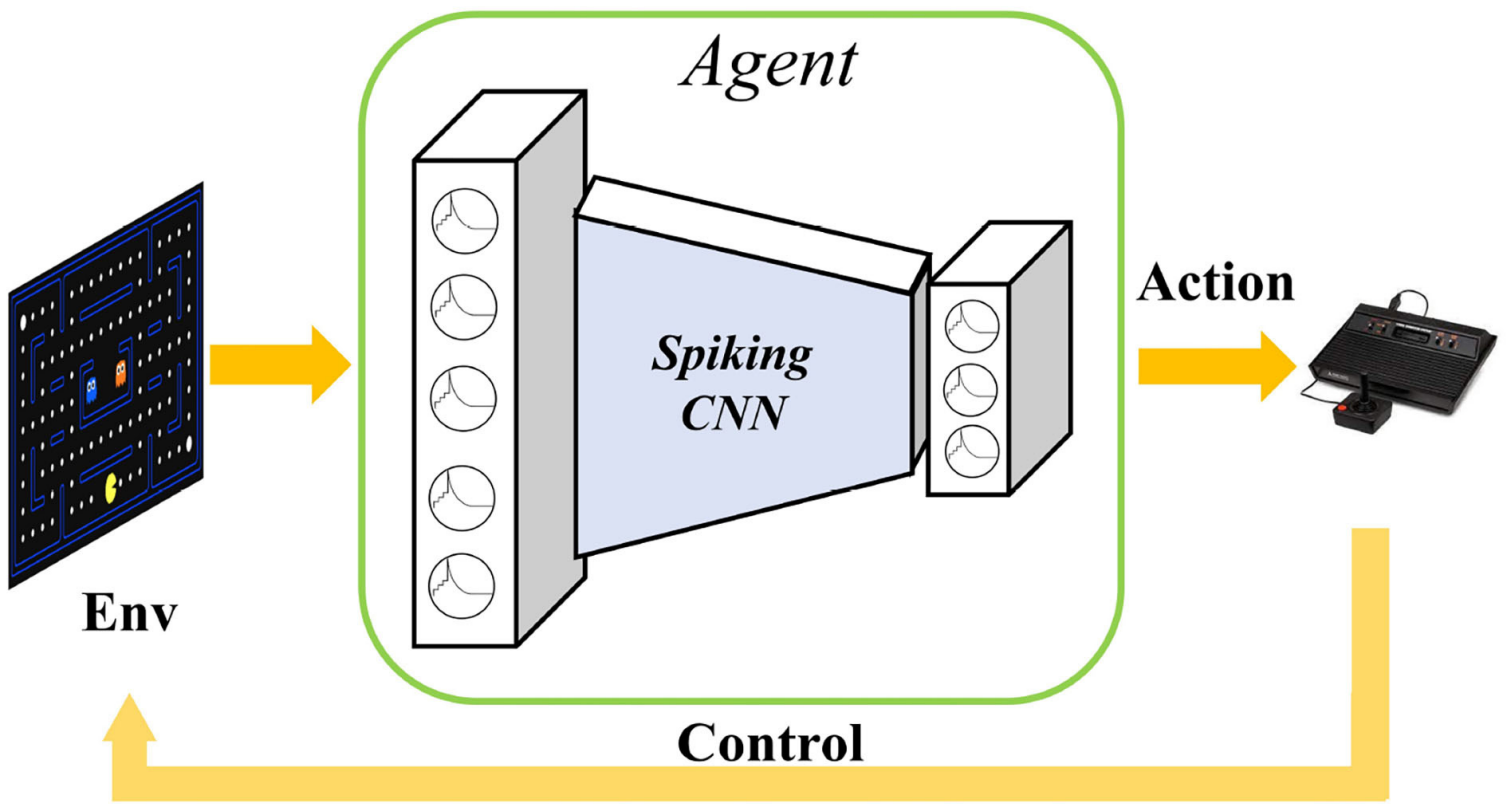
Playing Atari games with spiking neural networks. The raw original video image is input into the spiking convolutional neural networks to extract image features, and finally the action selection implemented by the SNN output layer. The selected action is used to control the activity of the agent in the environment to obtain more rewards.

Although there have been previous studies on the combination of SNNs and RL, most focus on robotic control problems with shallow networks and few neurons. Reward-modulated spike-timing-dependent plasticity (R-STDP) is used for training SNN to control robot keeping within the lane. Lele et al. proposed SNN central pattern generators (CPG) and leaned with stochastic reinforcement-based STDP to control hexapod walking (Lele et al., 2020). PopSAN (Tang et al., 2020) trained spiking actors with a deep critic network and validated on OpenAI gym continuous control benchmarks and autonomous robots. This work adopts actor-critic architecture and explores the combination of deep reinforcement learning and spiking neural networks. However, they only achieved implementing actor network with SNN, the critic for state-action value estimation was still using ANN. Therefore, they did not exploit the low-power advantage of implementing SNN. Because of the optimizing hardness and learning latency, agent based on SNN is challenging to be trained in reinforcement learning tasks. ANN-to-SNN conversion method (Rueckauer et al., 2017) is used to implement DQN with a spiking neural network (Patel et al., 2019; Tan et al., 2020). They first trained ANN-based DQN policy and then transferred the network weight to SNN, using SNN to plat Atari game as shown in Figure 1. Zhang et al. (2021) used knowledge distillation to train student SNN with a deep Q network teacher, but the student SNN does not being trained by the RL method with reward and does not interact with the environment.

Direct training of SNNs can obtain better performance advantages than ANNs to SNNs method and improve energy efficiency (Wu et al., 2019; Zheng et al., 2020). Although many successful cases of implementing a directly trained SNNs model in computer vision tasks, the direct training of SNNs in the deep reinforcement learning (DRL) model is facing more difficulty. One of the most important factors hindering the application of SNN in DRL is the disappearance of spike firing activity in deep spiking convolutional neural networks. The studies by Liu et al. (2021) and Chen et al. (2022) proposed direct training methods for spiking deep Q networks in RL tasks, but they do not deal with the vanishing problem in spiking activity in SDQN. In this study, we mathematically analyzed the problem of the disappearance of spiking signal features in SDQN and proposed a potential-based layer normalization (pbLN) method to train spiking deep Q networks directly. Experiments show that our study achieves better performance than SDQN based on the ANNs to SNNs method and other trained spiking DQN models. We summarize our contributions as follows:

- We analyze how the spiking mechanism influences information feature extraction in deep SNNs and found that the binary property of the spike hugely dissipates the variance and shifts the mean of network inputs. The pattern features of information are quickly vanishing in spiking deep Q networks.
- We propose the potential-based layer normalization method to keep the unique sensitivity of spiking neuron in deep Q networks.
- We construct a spiking deep Q network and implement it in gym Atari environments. The spiking deep Q network is directly trained with a surrogate function, and the experiments show that the pbLN improves the performance of SNN in RL tasks.

## 2. Methods

In this section, we introduce our study with three aspects. First, we construct a spiking deep Q network to estimate state-action value. Second, we analyze the feature vanishing in SNN and its influences on reinforcement learning. Third, we propose the potential-based layer normalization method and train the spiking deep Q network with a backpropagation algorithm.

### 2.1. Spiking deep Q network

**Figure 2:**
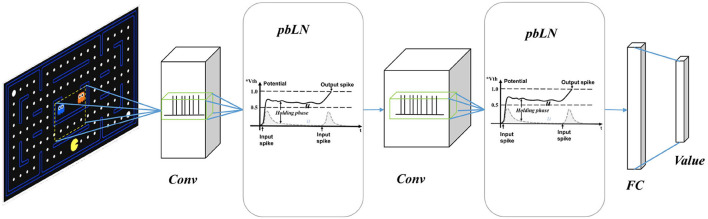
Spiking deep Q network with potential-based layer normalization. It has the same network structure as DQN, with three layers of convolution and two layers of full connection. The network outputs an estimate of the state-action value, which is used for the selection of actions and TD based learning.

In order to better reflect the characteristics of SNNs in the reinforcement learning environments, we construct our spiking Q networks as same as the DQN architecture shown in Figure 2. Three-layers spiking convolution neural networks process raw game screen images from gym Atari simulation to generate vision embedding. Then the vision embedding spike trains are processed by fully connected (FC) spiking neurons population to output control action. We use weighted spike integration to generate continuous real state-action values from discrete spikes.

The neuron model in the spiking Q Network is adapted from the leaky integrate-and-fire (LIF) model.


(1) $$\begin{array}{r} \tau \frac{du}{dt}=-u+x \end{array}$$

where *u* is membrane potential, and τ is the decay constant. *x* is postsynaptic potential (PSP). When the membrane potential exceeds thresholds *V*_*th*_, the neuron fires a spike, and the membrane potential is reset to *V*_*reset*_. Here, we use Heaviside step function *H* to implement the spiking procedure, and our model's neuron dynamic process can be described as follows:


(2) $$\begin{array}{r} u_{t+1}^{l}=u_{t}^{l}+\frac{1}{\tau }\left(-u_{t}^{l}+x_{t}^{l}\right) \end{array}$$

(3) $$\begin{array}{r} o_{t+1}^{l}=H\left(u_{t+1}^{l}-V_{th}\right) \end{array}$$

(4) $$\begin{array}{r} u_{t}^{l}=V_{reset},\;if\;o_{t}^{l}=1. \end{array}$$

Let $\alpha =1-\frac{1}{\tau }$ as decay factor of neuron potential, we get the clear format of Equation 2 as $u_{t+1}^{l}=\alpha u_{t}^{l}+\left(1-\alpha \right)x_{t}^{l}$. The $o_{t}^{l}$ represents the number *l* layer spike outputs. Neuron input *x*_*t*_ of convolutional layers in vision processing is a 2D convolution operation on the previous layer's spike features and can be written as:


$$\begin{array}{rc} x_{t}^{l}= & \;\left(w^{l}*o_{t}^{l-1}\right)\left[m,n\right]=\underset{j}{\sum }\underset{k}{\sum }w^{l}\left[j,k\right]o^{l-1} \end{array}$$

(5) $$\begin{array}{r} \left[m-j,n-k\right] \end{array}$$

where *w*^*l*^ is kernel weight of *l* convolutional layer. In the FC layer, *x*_*t*_ is the weighted sum of the previous layer spikes:


(6) $$\begin{array}{r} x_{t}^{l}=\underset{j}{\sum }w_{j}^{l}o_{t,j}^{l-1} \end{array}$$

State-action value is generated from the time-window mean value of weighted sum spikes output from FC layer as Equation (7) and *T* is the time-windows length of SNN simulation.


(7) $$\begin{array}{r} q_{i}=\frac{1}{T}\overset{T-1}{\underset{t=0}{\sum }}W_{i}\cdot O_{t} \end{array}$$

Unlike the ANN-SNN conversion based method or SNN-DNN hybrid training, our proposed model is directly optimized using the TD error about the network output with target values as


(8) $$L(w)=E[{\left(r+\gamma \underset{a}{max}Q(s^{\prime },a^{\prime },w)-Q(s,a,w)\right)}^{2}]$$

The proposed deep spiking Q network is directly trained by the Spatio-temporal Backpropagation (STBP) algorithm (Wu et al., 2018). For the final output weight *W*_*i*_, we have the derivatives:


(9) $$\begin{array}{r} \frac{\partial L}{W_{i}}=\frac{\partial L}{\partial Q}\frac{Q}{\partial W_{i}}=\frac{1}{T}\overset{T-1}{\underset{t=0}{\sum }}\delta^{L}O_{t} \end{array}$$

where δ^*L*^ is Q learning temporal difference


(10) $$\begin{array}{r} \delta^{L}=\frac{1}{2}\left[r+\gamma {max}_{a}Q\left(s^{\prime },a^{\prime },w\right)-Q\left(s,a,w\right)\right] \end{array}$$

The derivative temporal chain of the networks weight $w_{j}^{l}$ is written as:


(11) $$\begin{array}{l} \frac{\partial L}{\partial w_{j}^{l}}=\sum_{t=0}^{T-1}\frac{\partial L}{\partial o_{t+1}^{l}}\frac{\partial o_{t+1}^{l}}{\partial u_{t+1}^{l}}\frac{\partial u_{t+1}^{l}}{\partial w_{j}^{l}}\\ \;=\sum_{t=0}^{T-2}\frac{\partial L}{\partial o_{t+1}^{l}}\frac{\partial o_{t+1}^{l}}{\partial u_{t+1}^{l}}[\sum_{j}\left(1-\alpha \right)o_{t,j}^{l-1}+\alpha \frac{\partial u_{t}^{l}}{\partial w_{j}}] \end{array}$$

At the non-differentiable point of the neuron firing a spike, we use the surrogate function to approximate the derivative of *o*_*t*_ with respect to *u*_*t*_ as follows:


(12) $$\begin{array}{r} \frac{\partial o_{t+1}^{l}}{\partial u_{t+1}^{l}}=\frac{2\tau }{4+{\left(\pi \tau u_{t+1}^{l}\right)}^{2}} \end{array}$$

### 2.2. The feature information vanishing in spiking neural networks

In the process of deep network training, the change of network parameters will cause a change in the distribution of the network outputs, which is called *Internal Covariant Shift (ICS)* (Ioffe and Szegedy, 2015). Network parameters in ANN models are updated with training operations such as the gradient descent algorithm. Due to linear transformations and nonlinear activation in each layer, small changes in the low-level network layer will be amplified as the number of network layers deepens, and the network output will also change accordingly. Compared with ANN, SNN has more difficulty in processing information except for the ICS problem. First, unlike the linear transformation of ANN neurons, spiking neurons use kinetic equations to process input signal, which has a large nonlinear characteristic. Second, the spike outputs change the distribution of inputs severely, and the useful features of information are lost in deep layers. Thus, it needs more effort to solve the ICS problem in SNNs.

For *N* layers SNN, the proceeding procedure can be written as Algorithm 1. Because the spiking neuron model needs to accumulate the membrane potential in the whole simulation time window, the information is processed along both time and space dimensions. Considering the binary distribution of spikes *o*_*t*_, the means of spike *E*(*o*_*t*_) and variance *D*(*o*_*t*_) have below property $E[{\left(o_{t}\right)}^{2}]=E[o_{t}]\$and\$D(o_{t})=E(o_{t})(1-E(o_{t}))$.

**Algorithm 1:** Proceeding process of spiking deep Q networks.

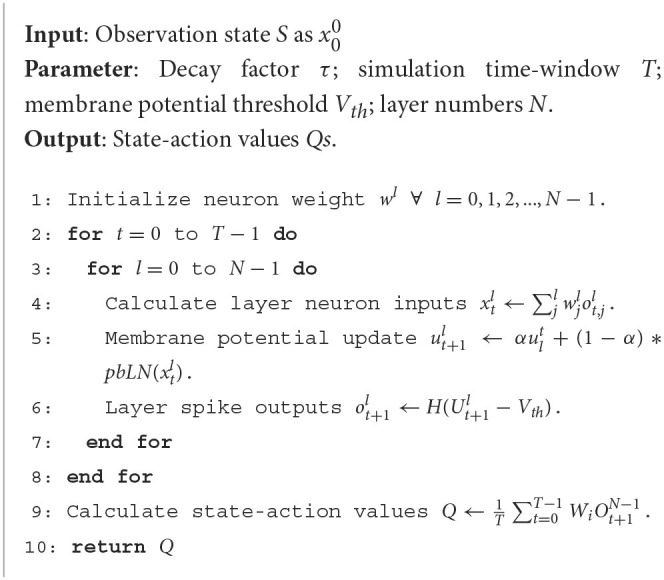

** LEMMA 1**. Let ψ(*i, j*) = (1−α)^2^α^2(*j*−*i*)^, synapse weight *W* is randomly initialized with *E*(*W*) = 0 and is independent with *o*_*t*_, the variance of neuron potential is calculated as


(13) $$D(u_{t+1}^{l})=D(W^{l})\sum_{i=0}^{t}\psi (i,t)E[o_{i}^{l-1}]$$

Supposing the neuron in layer *l* fired the first spike after *t* time steps, we denote the variance of neuron membrane potential at time *t*+1 and layer *l* as Equation 15. Lemma 1 shows the relationship of neuron membrane potential *u*_*t*_ with previous inputs spike history and synaptic weight. Additionally, the presynaptic spike inputs' effect decays with the time factor α for ψ(*i, j*)∈(0, 1]. Refer to the proofs in Supplementary material for details.

** THEOREM 1**. Let $\varepsilon =\frac{D(W^{l})}{2V_{th}^{2}}$, the neuron in layer *l* firing spikes with:


(14) $$E(o_{t+1}^{l})\leq \varepsilon E[\sum_{i=1}^{t}o_{i}^{l-1}]$$

ε is the signal loss ratio transmitted by spiking neural networks. Additionally, if ε < 1, the mean of neuron's spikes *E*(*o*_*t*_) tends to zero with the increase of the number of layers. The variance of spikes *D*(*o*_*t*_) is also decreasing, which results in neuronal firing spikes vanishing rapidly and the deep layer of SNNs is very prone to no spiking activity. This problem makes deep SNNs lose signal features in information processing. In addition, in deep spiking convolutional neural networks, which use local connections and sharing weight operations, the spike signal disappearance problem is more prominent. It makes it hard for the deep SCNN to be directly trained and weakens the performance of SNN models.

### 2.3. Potential based layer normalization

According Equation (16), the problem of spike information vanishing in SNNs can be alleviated by initializing synapse weights with greater distributional variance or setting the spiking neuron model with a little potential threshold. But increasing *D*(*W*) will damage performance and make converging the model difficult. Besides, too small threshold potential will make neurons too active to distinguish useful information. To solve this contradiction, some works using the potential normalization methods in spiking neural networks, such as NeuNorm (Wu et al., 2019) using auxiliary neurons to add spikes together and proposing inputs with scalar norm, and tdNorm (Zheng et al., 2020) extending batch normalization to time dimensions.

But these methods are suitable for supervised learning tasks such as image classification or object detection because, in those tasks, the SNNs are trained with batched data inputs. Compared with supervised learning, the environment information of reinforcement learning is more complex. First, in RL tasks spike vanishing problem of deep SNN models is quite serious. For example, we counted each layer's spiking deep Q network firing activity distribution when applied to Atari games. The statistical evidence in the Result section shows that the SDQN suffers serious spiking information reduction in deep layers. Second, unlike supervised learning, SNN agents in RL have no invariant and accurate learning labels and need to interact with the environment to collect data and reward information. The hysteresis of learning samples makes the SDQN model unable to effectively overcome the drawbacks caused by the disappearance of spike signals in output layers. Third, the input format in the RL task is not batched, so the normalization methods used in supervised learning can not be applied to SDQN.

In this study, we propose a potential-based layer normalization method to solve the spike activity vanishing problem in SDQN. We apply the normalization operation methods on PSP *x*_*t*_ in convolution layers. The previous layers' spikes are processed as Equation 5 and further normalized as follows:


(15) $$\begin{array}{r} \hat{x_{t}}=\frac{x_{t}-{\bar{x}}_{t}}{\sqrt{\sigma_{x_{t}}+\epsilon }} \end{array}$$

(16) $$\begin{array}{r} {\bar{x}}_{t}=\frac{1}{H}\overset{H}{\underset{i=1}{\sum }}x_{t,i} \end{array}$$

(17) $$\begin{array}{r} \sigma_{x_{t}}=\frac{1}{H}\sqrt{\overset{H}{\underset{i=1}{\sum }}{\left(x_{t,i}-{\bar{x}}_{t}\right)}^{2}} \end{array}$$

where in convolution layer *H* = *C*× *H*× *W* with *C* for channels number, and [*H, W*] is the feature's shape in each channel.

PSP *x*_*t*_ is normalized into distribution with zero mean and one variance is shown as Equation 17. This normalization method is different from NeuNorm and tdNorm. NeuNorm applied normalization operation on neuron spikes *o*_*t*_. Additionally, tdNorm used batch normalization method on the time dimension, which needs to calculated [*x*_*t*+1_, *x*_*t*+2_, …, *x*_*t*+*T*_] in advance.

**Figure 3:**
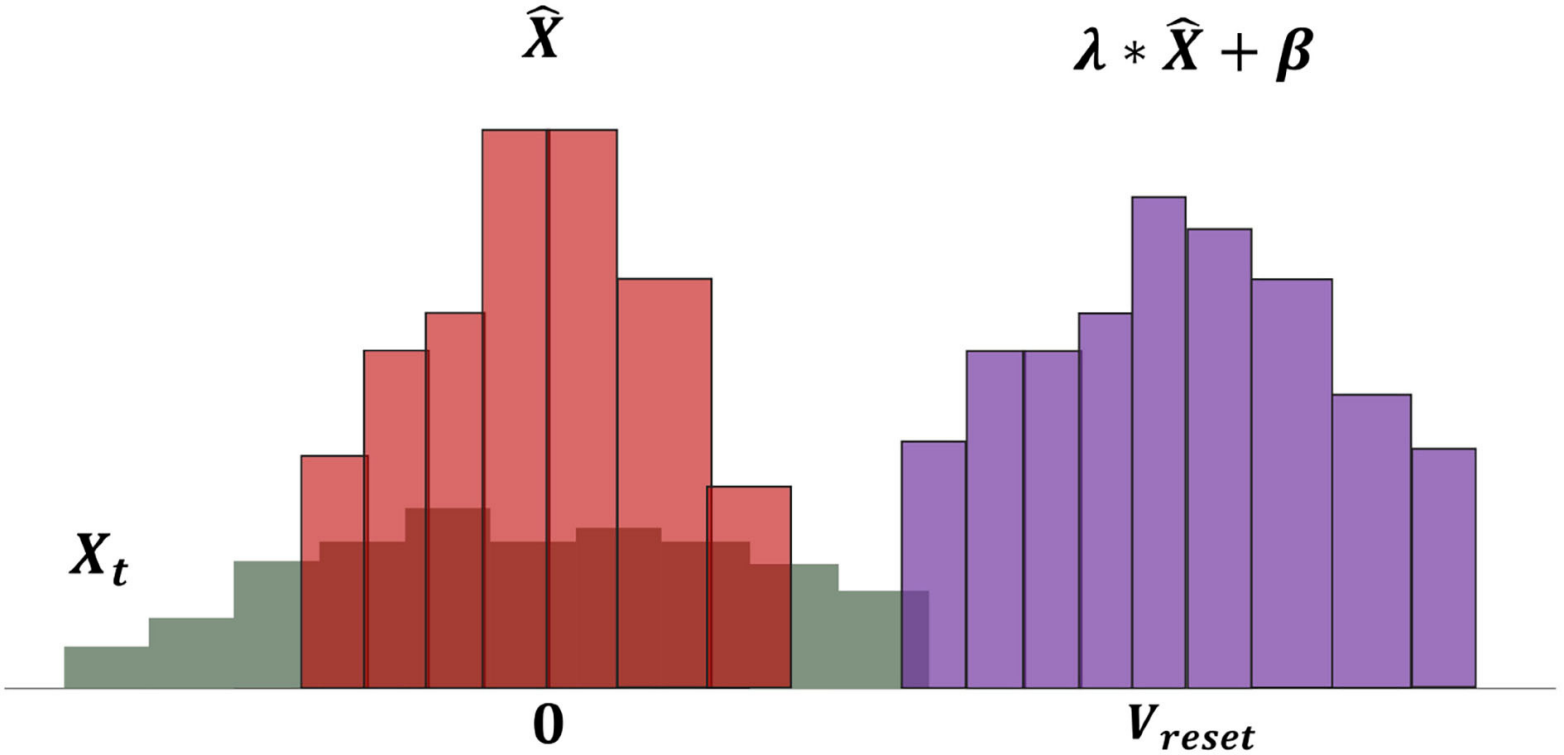
Operations of neural potential-based layer normalization. The bars in the figure represent the distribution of PSP potential. The gray bars show the original PSP potential *X*, and it is first normalized to zero means and one variance $\hat{X}$ magenta bars. The final shift factor β and scale factor λ make the PSP potential suiting to spiking neurons.

Normalizing *x*_*t*_ will weaken the characteristic information in the feature maps, and the zero means and one variance do not suit the spiking neuron. Thus, we adapted the LIF neuron model as:


(18) $$\begin{array}{r} u_{t+1}=\alpha u_{t}+\left(1-\alpha \right)\left[\lambda_{t}*{\hat{x}}_{t}+\beta_{t}\right] \end{array}$$

(19) $$\begin{array}{r} \lambda_{0}=V_{th}-V_{reset},\;\beta_{0}=V_{reset} \end{array}$$

where λ_*t*_ and β_*t*_ are learnable parameters, which are initialized at the beginning as Equation 21. The process of pbLN changes the distribution of neural PSP and is depicted in Figure 3. The parameter λ_*t*_ has the same effect of increasing *D*(*W*), and β_*t*_ plays a role in the dynamic firing threshold. By separating the learnable parameters, the SNN model avoids the oscillation of the learning process caused by increasing *D*(*W*) and the over-discharge of neurons caused by the threshold being too small, which reduces the information processing capability of the model.

**Figure 4:**
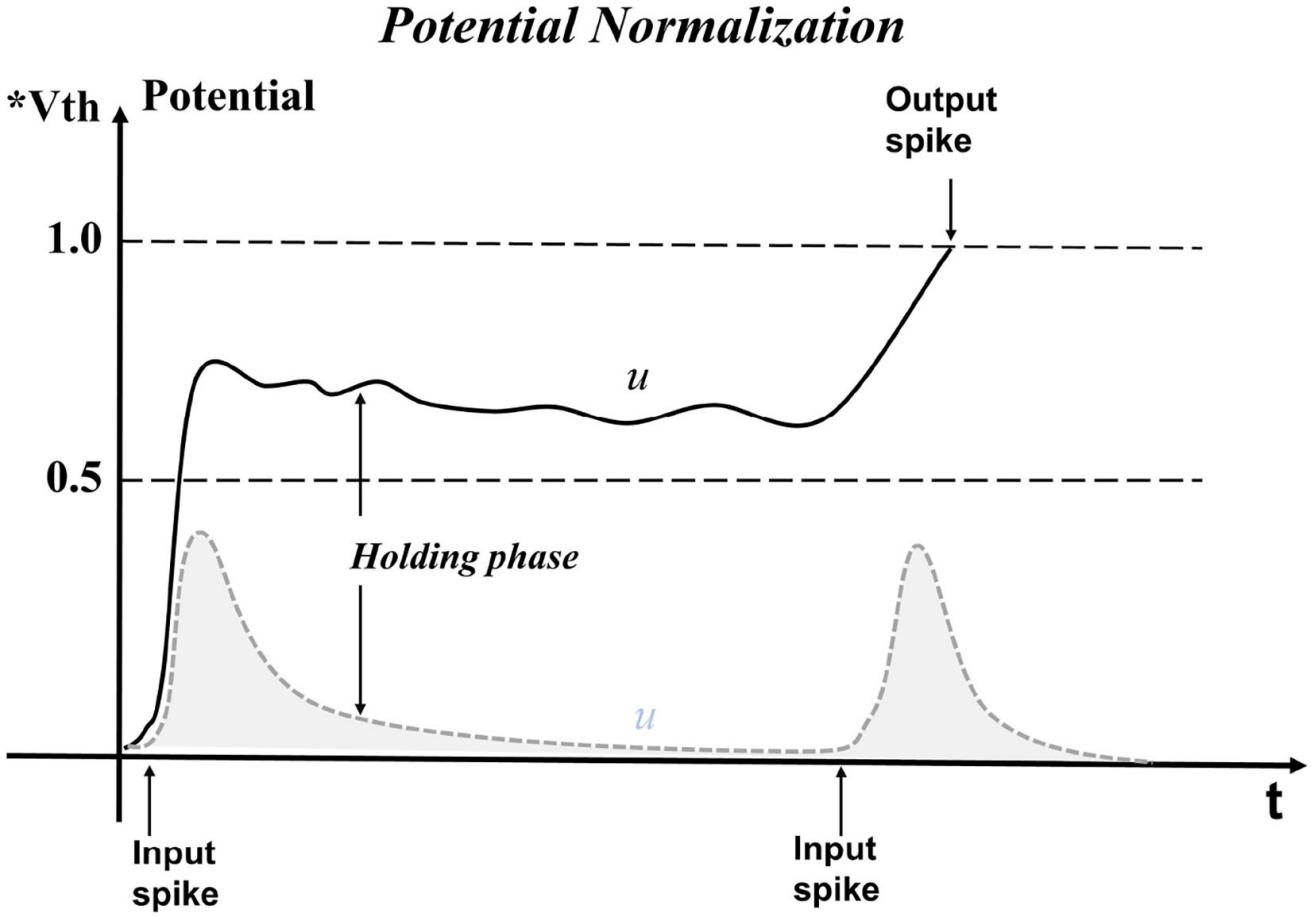
Neuron potential is maintained by the normalization method. The gray dotted line is the change of membrane potential of neurons without normalization when receiving spike inputs. Additionally, the solid black line is neuron potential changed by normalization. Neurons are difficult to fire when the time interval of external stimulation is relatively large. Instead, with normalization operations, the membrane potential is affected by the neighbor neurons, and the leakage trend will slow down, which increases the probability of neurons firing spikes.

The effect of pbLN on membrane potential is shown in Figure 4. When the spike signal of the previous layer is input, the membrane potential begins to rise. Compared with the LIF model, neurons with the function of pbLN are affected by neighbors and can hold the membrane potential values so that the neuron can fire a spike as long as it receives little input in the future. This puts the neuron in an easy-to-fire state where it can process long-time interval signals and reduces the loss of features when passing on the input information.

## 3. Results

PL-SDQN is a spiking neural network model based on LIF neurons and has the same network structure as traditional DQN. It contains three convolutional layers with a “c32k8-c64k4-c64k3” neural structure. The hidden layers are fully connected with 512 neurons, and the output is ten values as the weighted summation of the outputs of the hidden layer. We directly trained PL-SDQN on reinforcement learning tasks. The results show that spiking deep Q networks combined with the potential-based layer normalization method can achieve better performance on Atari games than traditional DQN and ANN-to-SNN conversion methods.

### 3.1. Statistic evidence of spiking activity reduction in deep layers

We counted each layer's firing spikes of SDQN to show the deep layer spike vanish phenomenon and the promoting effect of the pbLN method. The SDQN model is initialized by random synaptic weight and then used to play the Atari game. We calculated the ratio of neurons with firing activity to each layer's total number of neurons.

**Figure 5:**
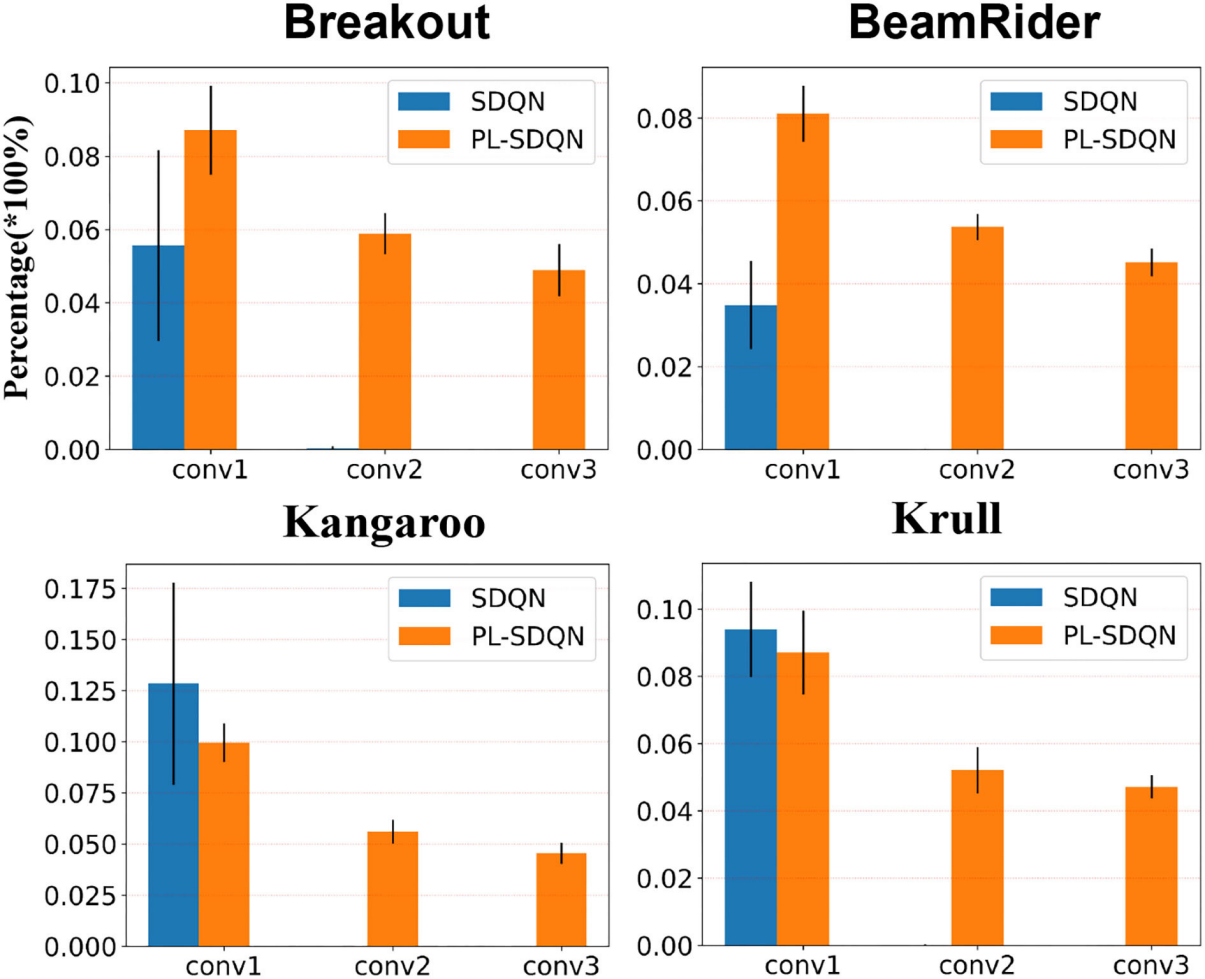
Fire rates on different convolutional layers of SDQN. The blue bars depict the spike fire rate in SDQN without normalization. Additionally, the orange bars are for our model PL-SDQN. The black vertical line on top of each bar is the SD of 10 experimental data.

We tested each game ten times and counted the firing rate of each layer. These experiments' average and SD are shown in Figure 5. The results show that convolution layers in SDQN are difficult to transfer spiking activities. Spikes from the first layer (conv1) are rarely transmitted to the next layer. There is almost no spike firing activity in the second (conv2) and third (conv3) layers.

Compared with the vanishing problems in SDQN, the proposed pbLN method improves the deep layer spiking activities. The bottom rows in Figure 5 show that the pbLN method not only increases the first layer's activity to make later layers fire more spikes it also improves the inner sensitivity of each layer of the network to spike inputs.

### 3.2. Performance and analysis of Atari games

**Figure 6:**
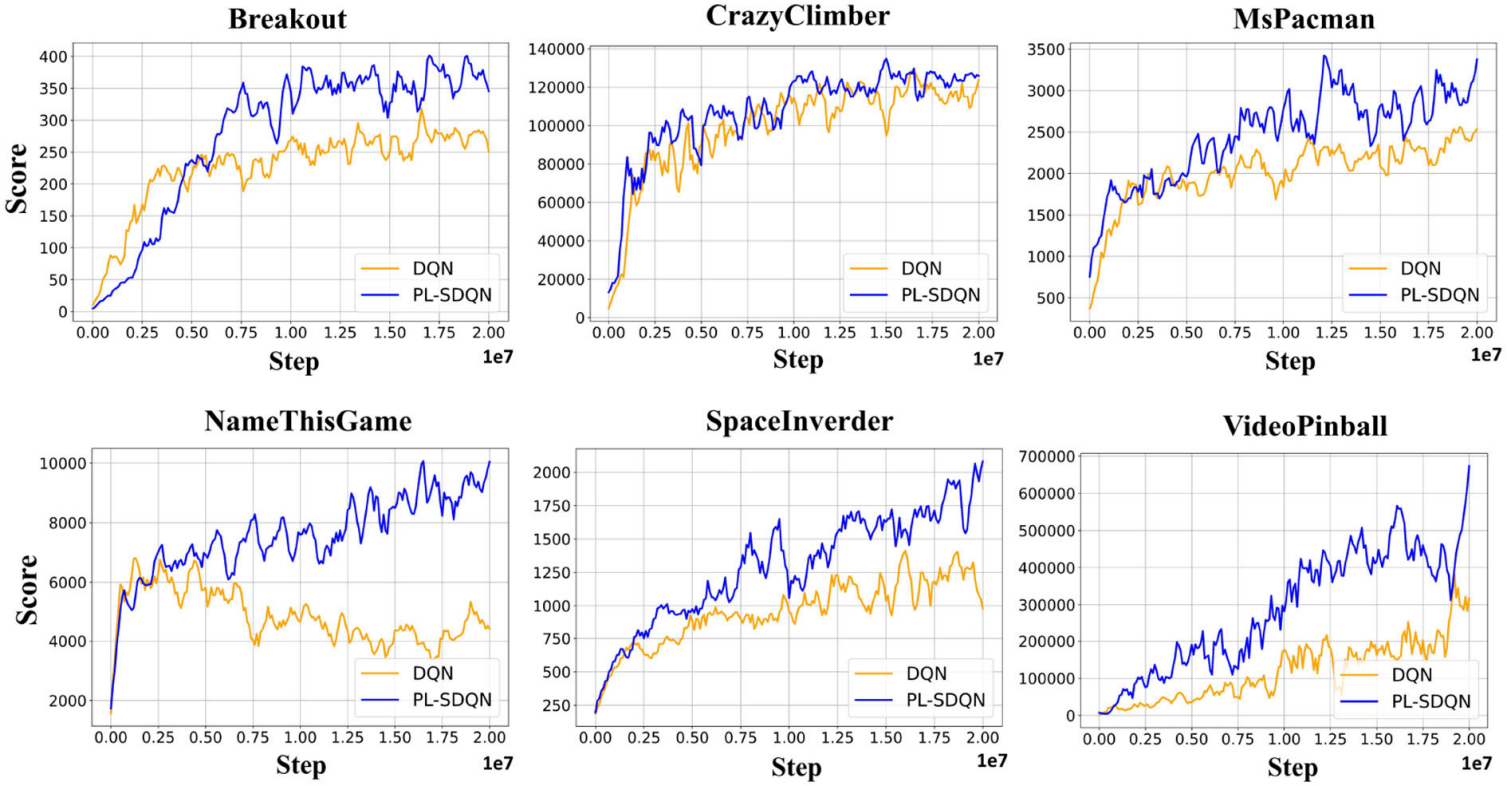
PL-SDQN performance on Atari games. The learning curves in the figure show that our PL-SDQN model achieves faster and better performance than the original DQN benchmarks. Although in the Breakout game, the DQN model learns faster at the beginning, our model catches up with it quickly and achieves better performance at the end. We smooth each curve with a moving average of 5 for clarity.

We compared our model with the vanilla DQN model and ANN-SNN conversion based SDQN model, and the performance are obtained on 16 Atari games. All models are trained directly with the same settings and optimized by Adam's methods as Supplementary Table S1. The ANN-SNN conversion based SDQN implements the same method as the state-of-the-art works proposed by Li and Zeng (2022) with simulation time window *T*_*con*_ = 256. Additionally, the PL-SDQN is directly trained by the backpropagation method with simulation times set as *T* = 16. We train all of the models for 20 million frame steps. We conducted ten tests and recorded the mean and SD of scores. The results are shown in Figure 6 and Table 1.

**Table 1:** Details of Atari game experiments.

	**DQN**			**ANN-SNN conversion**			**PL-SDQN**	
**Game**	**Score**	**±std**	**(Score%)**	**Score**	**±std**	**(Score%)**	**Score**	**±std**	**(Score%)**
Atlantis	3,049,750.0	161,861.4	(5.31%)	304,6920.0	13,48,868.6	(44.27%)	**32,67,760.0**	86,339.7	(2.64%)
BeamRider	10,423.2	2,245.1	(21.54%)	10,449.0	262,0.4	(25.08%)	**11,480.4**	33,42.4	(29.11%)
Boxing	99.3	0.9	(0.91%)	98.6	3.2	(3.25%)	**99.5**	0.9	(0.90%)
Breakout	343.1	41.3	(12.04%)	352.2	64.7	(18.37%)	**427.7**	140.1	(32.76%)
CrazyClimber	1,39,420.0	11,530.6	(8.27%)	12,8,380.0	23,239.8	(18.10%)	**14,7,950.0**	30,6,32.1	(20.70%)
Gopher	**38,662.0**	28,245.6	(73.06%)	22,438.0	10,076.7	(44.91%)	24,064.0	12,3,55.3	(51.24%)
Jamesbond	14,45.0	15,72.0	(108.79%)	14,20.0	190.0	(13.38%)	**146,0.0**	215,0.1	(147.27%)
Kangaroo	12,680.0	208.8	(1.65%)	13,850.0	113,2.5	(8.17%)	**145,00.0**	845.0	(5.83%)
Krull	10,271.0	1,365.5	(13.29%)	10,923.0	513.0	(4.70%)	**11,807.0**	568.2	(4.81%)
MsPacman	2,964.0	711.7	(24.01%)	36,91.0	434.8	(11.78%)	**4,077.0**	12,92.5	(31.70%)
NameThisGame	7,732.0	1,289.2	(16.67%)	8115.0	1,702.1	(20.97%)	**12,202.0**	22,10.7	(18.12%)
RoadRunner	1,310.0	764.8	(58.38%)	1,072.0	329.2	(19.34%)	**51,930.0**	47,14.4	(9.08%)
SpaceInvaders	1,728.5	461.6	(26.71)	176,0.0	483.5	(27.47%)	**24,33.5**	574.7	(23.62%)
StarGunner	53,050.0	1342.5	(2.53%)	55,910.0	12,796.9	(22.89%)	**63,560.0**	40,64.7	(6.40%)
Tutankham	262.0	28.9	(11.03%)	254.5	55.4	(21.77%)	**271.5**	70.4	(25.93%)
VideoPinball	5,07,442.5	327,1,89.1	(64.48%)	55,2917.6	20,0852.5	(36.33%)	**67,35,53.0**	100,66.2	(1.49%)

The vanilla DQN, ANN-SNN conversion based SDQN and our proposed model PL-SDQN are compared. We test these models for 10 rounds and record raw scores' mean and standard deviation (STD).

The best scores of each game are highlighted by bold values.

PL-SDQN model that we proposed achieves better performance than vanilla DQN and conversion based SDQN model. The data in Table 1 shows that our model has achieved performance advantages over the other two methods in 15 games. Additionally, the curves in Figure 6 show that our method achieves faster and more stable learning than the vanilla DQN.

### 3.3. Experiments on potential normalization effects

In order to show the improvement of our proposed pbLN method on the spiking deep Q model, we compared PL-SDQN with other directly trained SDQN models in articles by Liu et al. (2021) and Chen et al. (2022). Because the other works compared with different DQN backbones, we recorded the promotion rate of SDQN model scores for the special DQN methods the authors compared in their article.

The result in Table 2 shows that our PL-SDQN model is more robust on different games and achieves better performance than other SDQN methods. Compared with the SNN model in Chen et al. (2022), the PL-SDQN has better generalization and robustness in more experiments for successfully surpassing DQN benchmarks on 14 games out of a total of 15 games tested.

**Table 2:** The comparison of our PL-SDQN model with state-of-art spiking deep Q networks.

	**SDQN(Liu2021)**	**SDQN(Chen2022)**	**PL-SDQN(Ours)**
**Game**	**(DQN%)**	**(DQN%)**	**(DQN%)**
Atlantis	98.79	84.24	**107.15**
BeamRider	97.48	99.57	**110.14**
Boxing	99.17	**298.23**	100.20
Breakout	90.86	**144.38**	124.66
CrazyClimber	102.82	**109.79**	106.12
Gopher	95.78	**148.96**	62.24
Jamesbond	**127.57**	113.92	101.04
Kangaroo	**214.49**	94.56	114.35
Krull	106.77	28.69	**137.55**
NameThisGame	98.85	152.41	**157.81**
RoadRunner	89.72	917.26	**3,964.12**
SpaceInvaders	80.5	106.85	**140.79**
StarGunner	112.96	**153.73**	119.81
Tutankham	103.90	**280.48**	103.63
VideoPinball	87.01	**159.98**	132.73
Total ≥ 100 %	6/15	11/15	**14/15**

To show the SNN advantage, we record the percentage as SDQN/DQN * 100%. The best scores of each game are highlighted by bold values.

We analyze that our model has an advantage in the test game because the spiking activity vanishing in deep layers of SNN reduces the performance of the SDQN model. The proposed pbLN method can well counteract the impact of the input signal change on the model's performance to improve the ability to spike neural networks in the reinforcement learning task. Unlike PL-SDQN, the SDQN method based on ANN-SNN conversion faces the problem of spike accuracy and requires a long simulation process. The performance of ANN-SNN conversion SDQN is challenging to surpass the original ANN model. The other directly trained SDQN models compared in Table 2 do not focus on the spike information vanishing problem in spiking neural networks. Although the layer normalization method weakens the specificity of the convolutional layer channels, it helps boost the spiking convolution neural network's performance for increasing neuronal activity.

The primary computational consumption of potential-based layer normalization is concentrated in the features mean and variance calculation. The computational complexity of mean and variance operation is O(X) and $O\left(X^{2}\right)$ severally. Thus, we can get the pbLN method's computational complexity as $O\left(X^{2}\right)$. Except for the SDQN model, the pbLN method can be used for other SNN models that do not process data in large batch format, such as recurrent spiking neural networks and SNNs for robot control tasks.

## 4. Discussion and conclusion

In this study, we directly trained the deep spiking neural networks on the Atari game reinforcement learning task. Because of the characteristics of discrete bias and the hard optimization problem, spiking neural network is difficult to apply to the reinforcement learning field in complex scenarios. We mathematically analyze why spiking neural networks are difficult to generate firing activity and propose a potential based layer normalization method to increase spiking activity in deep layers of SNN. This method can increase the firing rate of the deep spiking neural network so that the input information features can be transferred to the output layer. Additionally, the experiment results show that compared with vanilla DQN and ANN-SNN conversion based SDQN methods, our PL-SDQN model achieves better task performance. Besides, our model has better generalization and robustness compared to other directly trained SDQN methods on Atari game reinforcement learning tasks.

## Data availability statement

The original contributions presented in the study are included in the article/Supplementary material, further inquiries can be directed to the corresponding author. Source code can be found in https://github.com/BrainCog-X/Brain-Cog/tree/main/examples/decision_making/RL.

## Author contributions

YS wrote the code, performed the experiments, analyzed the data, and wrote the manuscript. YZ proposed and supervised the project and contributed to writing the manuscript. YL participated in helpful discussions and contributed to part of the experiments. All authors contributed to the article and approved the submitted version.

## Funding

This study was supported by the National Key Research and Development Program (Grant No. 2020AAA0104305), the Strategic Priority Research Program of the Chinese Academy of Sciences (Grant No. XDB32070100), and the National Natural Science Foundation of China (Grant No. 62106261).

## Conflict of interest

The authors declare that the research was conducted in the absence of any commercial or financial relationships that could be construed as a potential conflict of interest. The handling editor Z-GH declared a shared affiliation with the authors at the time of review.

## Publisher's note

All claims expressed in this article are solely those of the authors and do not necessarily represent those of their affiliated organizations, or those of the publisher, the editors and the reviewers. Any product that may be evaluated in this article, or claim that may be made by its manufacturer, is not guaranteed or endorsed by the publisher.
