# Supplementary material for: Solving the spike feature information vanishing problem in spiking deep Q network with potential based normalization
Source: Front Neurosci. 2022 Aug 25;16:953368. doi: 10.3389/fnins.2022.953368 (PMC9453154; doi:10.3389/fnins.2022.953368)
Supplement: Supplementary file 1 [file Data_Sheet_1.PDF]

Supplementary Material:Solving the Spike Feature Informationg Vanishing Problem in Spiking Deep Q Network with Potential Based Normalization

1 PROOFS

PROOF. Lemma 1

In this work the synapse weight value is sampled from uniform distribution $U(-k,k)$, k is constant and related with inputs signal dimension. We get the variance of postsynaptic potential x_t as

$$\begin{aligned}\mathbb{D}(x_t^l) &= \mathbb{D}(W^l o_t^{l-1}) \\ &= \mathbb{E}[(W^l)^2(o_t^{l-1})^2] - \mathbb{E}[(W^l)]\mathbb{E}[(o_t^{l-1})] \\ &= \mathbb{E}[(W^l)^2]\mathbb{E}[(o_t^{l-1})^2] \\ &= [\mathbb{D}(W^l) + \mathbb{E}^2(W^l)]\mathbb{E}(o_t^{l-1}) \\ &= \mathbb{D}(W^l)\mathbb{E}(o_t^{l-1})\end{aligned} \tag{S1}$$

Then, we use the recursive method to deduce the relationship between the membrane potential u_t variance and the presynaptic spikes:

$$\begin{aligned}\mathbb{D}(u_{t+1}^l) &= \mathbb{D}(\alpha u_t^l + (1-\alpha)x_t^l) \\ &= \alpha^2\mathbb{D}[(u_t^l)^2] + (1-\alpha)^2\mathbb{D}[x_t^l] \\ &= \alpha^2\mathbb{D}[(u_t^l)^2] + (1-\alpha)^2\mathbb{D}(W^l)\mathbb{E}(o_t^{l-1}) \\ &= \mathbb{D}(W^l)\sum_{i=0}^{t}\mathbb{E}[o_i^{l-1}]\alpha^{2(t-i)}(1-\alpha)^2\end{aligned} \tag{S2}$$

PROOF. Theorem 1

The membrane potential u_{t+1} accumulates the former t times input spikes o_t. And the synapse weight is initialized $\mathbb{E}(W^l) = 0$

$$
\begin{aligned}
\mathbb{E}(u_{t+1}^{l}) &= \alpha\mathbb{E}(u_{t}^{l}) + (1-\alpha)\mathbb{E}(x_{t}^{l}) \\
&= \sum_{i=0}^{t} \alpha^{t-i}(1-\alpha)\mathbb{E}(x_{i}^{l}) \\
&= \sum_{i=0}^{t} \alpha^{t-i}(1-\alpha)\mathbb{E}(W^{l})\mathbb{E}(o_{i}^{l}) \\
&= 0
\end{aligned} \tag{S3}
$$

According to the Chebyshev's inequality:

$$
\begin{aligned}
\mathbb{E}(o_{t+1}^{l}) &= P(u_{t+1}^{l} > V_{th}) \\
&= \frac{1}{2}P(|u_{t+1}^{l} - \mathbb{E}(u_{t+1}^{l})| > V_{th}) \\
&\leq \frac{\mathbb{D}(u_{t+1}^{l})}{2V_{th}^{2}}
\end{aligned} \tag{S4}
$$

In spiking neural model time constant $\tau \geq 1.0$, the range of decay factor $\psi(i,j) \in [0,1)$ then we get

$$
\begin{aligned}
\mathbb{E}(o_{t+1}^{l}) &\leq \frac{\mathbb{D}(u_{t+1}^{l})}{2V_{th}^{2}} \\
&\leq \frac{\mathbb{D}(W^{l})}{2V_{th}^{2}} \sum_{i=0}^{t} \psi(i,t)\mathbb{E}[o_{i}^{l-1}] \\
&\leq \frac{\mathbb{D}(W^{l})}{2V_{th}^{2}} \mathbb{E}[\sum_{i=0}^{t} o_{i}^{l-1}]
\end{aligned} \tag{S5}
$$

Parameters	Value	Description
τ	2	Membrane time constant
V_{th}	1.0	Threshold potential
V_{reset}	0.0	Reset potential
T	16	Simulation time window
γ	0.99	Discount factor of DQN
T_{con}	256	Conversion time window
lr	0.0001	Learning rate

Table S1. Settings of models and experiments.
